# Transferable Classical Force Field for Pure and Mixed Metal Halide Perovskites Parameterized from First-Principles

**DOI:** 10.1021/acs.jcim.1c01506

**Published:** 2022-05-16

**Authors:** Juan Antonio Seijas-Bellido, Bipasa Samanta, Karen Valadez-Villalobos, Juan Jesús Gallardo, Javier Navas, Salvador R. G. Balestra, Rafael María Madero Castro, José Manuel Vicent-Luna, Shuxia Tao, Maytal Caspary Toroker, Juan Antonio Anta

**Affiliations:** † Área de Química Física, Universidad Pablo de Olavide, Seville, 41013, Spain; ‡ Department of Materials Science and Engineering, Technion−Israel Institute of Technology, Haifa, 3200003, Israel; ¶ Departamento de Química Física, Facultad de Ciencias, Universidad de Cádiz, Puerto Real, Cádiz E-11510, Spain; § Instituto de Ciencia de Materiales de Madrid, Consejo Superior de Investigaciones Científicas (ICMM-CSIC) c/Sor Juana Inés de la Cruz 3, Madrid, 28049, Spain; ∥ Materials Simulation and Modelling, Department of Applied Physics, Eindhoven University of Technology, P.O. Box 513, Eindhoven, 5600MB, The Netherlands

## Abstract

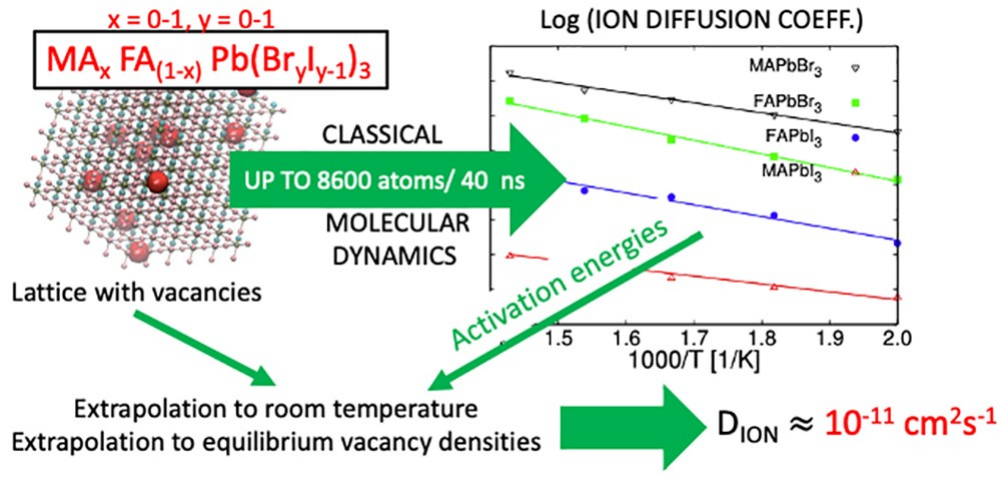

Many key features in photovoltaic perovskites occur in relatively long time scales and involve mixed compositions. This requires realistic but also numerically simple models. In this work we present a transferable classical force field to describe the mixed hybrid perovskite MA_*x*_FA_1–*x*_Pb(Br_*y*_I_1–*y*_)_3_ for variable composition (∀*x*, *y* ∈ [0, 1]). The model includes Lennard-Jones and Buckingham potentials to describe the interactions between the atoms of the inorganic lattice and the organic molecule, and the AMBER model to describe intramolecular atomic interactions. Most of the parameters of the force field have been obtained by means of a genetic algorithm previously developed to parametrize the CsPb(Br_*x*_I_1–*x*_)_3_ perovskite (Balestra et al. *J. Mater. Chem. A*. **2020**, DOI: 10.1039/d0ta03200j). The algorithm finds the best parameter set that simultaneously fits the DFT energies obtained for several crystalline structures with moderate degrees of distortion with respect to the equilibrium configuration. The resulting model reproduces correctly the XRD patterns, the expansion of the lattice upon I/Br substitution, and the thermal expansion coefficients. We use the model to run classical molecular dynamics simulations with up to 8600 atoms and simulation times of up to 40 ns. From the simulations we have extracted the ion diffusion coefficient of the pure and mixed perovskites, presenting for the first time these values obtained by a fully dynamical method using a transferable model fitted to first-principles calculations. The values here reported can be considered as the theoretical upper limit, that is, without grain boundaries or other defects, for ion migration dynamics induced by halide vacancies in photovoltaic perovskite devices under operational conditions.

## Introduction

Metal halide perovskites (MHP) have emerged as one of the most studied semiconductors due to their excellent optoelectronic properties.^2,3^ This is evidenced by the rapid development of perovskite solar cells (PSCs) with a record certified photoconversion efficiency of 23.7%, similar to those of silicon cells.^4^ Nonetheless, industrial application of PSCs is critically hampered by instability issues, including intrinsic, environmental, and operational factors. Instability is attributed to several chemical and dynamical processes that occur at very distinct time scales, like ionic rearrangements and physical and chemical interactions in the bulk and at interfaces with contact layers. These phenomena cause current–voltage hysteresis and, ultimately, device degradation.

**Figure 1:**
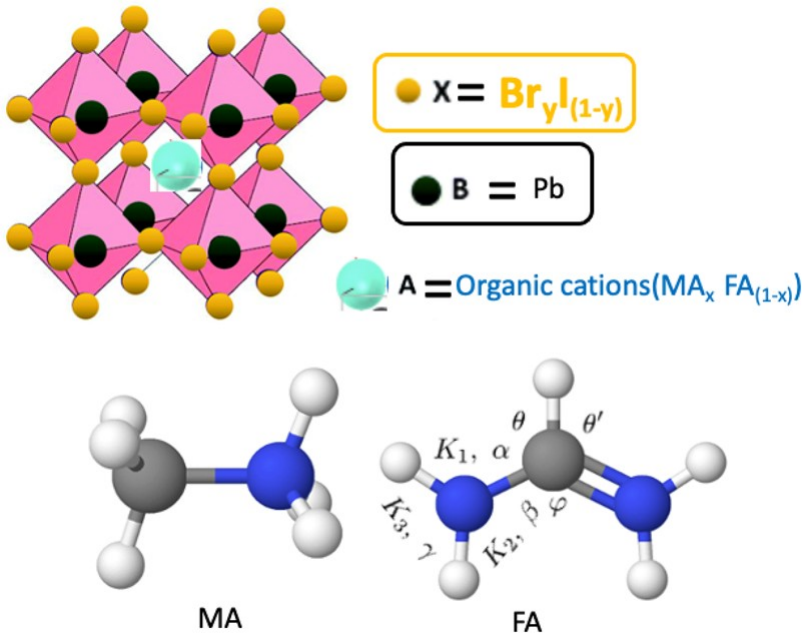
Top: crystalline structure of metal halide perovskites. Bottom: Methylammonium (MA, left) and formamidinium (FA, right) ions. The atoms included in the molecules are C (gray), N (blue), and H (white). Useful notation has been included in the FA molecule. (Figure created using https://molview.org.).

It has been proven that the inclusion of organic molecules as the A ion in the ABX_3_ formula of the perovskites increases the efficiency and the stability of the cells. Promising results have been recently reported for FAPbI_3_ solar cells, in which the efficiencies obtained reach values larger than 25%.^5−7^ However, the structural phase that allows for the absorption of photons and the creation of electron–hole pairs, the α-black phase, is unstable, and rapidly decays to the δ-yellow phase, useless for solar cell applications.^6,8^ A possible solution for that is to mix different ions and molecules in the same active layer, like the cesium ion and the methylammonium (MA) and formamidinium (FA) molecules (Figure 1), which significantly increases its stability without paying a high cost in efficiency.^9^

The combination of A = Cs, MA, FA; B = Pb; X = Br, I is well-known to researchers working in perovskite solar cells (PSCs). Many theoretical and experimental papers reported the structural parameters and the different properties of this kind of systems. Nevertheless, most of the theoretical studies have been performed using Density Functional Theory (DFT) calculations that imply working with a small number of atoms and allow for very short time dynamics simulations. Some phenomena that have been experimentally observed and described in these mixed supercells, such as ionic diffusion and segregation, cannot be modeled using first-principles techniques. This is one of the reasons that leads us to think that a classical model for hybrid perovskites would be very useful, since a parameter such as the diffusion coefficient would be easily accessible through a classical molecular dynamics (CMD) simulation.

The strategy of developing and using quantum mechanically derived force fields for functional materials is not new and has been applied to model noncovalent interactions in proteins.^10,11^ More recently, this kind of method has been explored and applied to functional materials such as organic LEDs by Odinokov and co-workers.^12^ Prampolini et al.^13^ used quantum mechanically derived force fields to predict self-assembly of organic molecules.

In the specific case of perovskites, two classical force fields (FFs) developed in the University of Cagliari by Mattoni and co-workers for the MAPbBr_3_^14^ and the MAPbI_3_^15^ reproduce accurately the behavior of these two compounds. Handley and Freeman^16^ also devised a FF for MAPbI_3_ by fitting ab initio and experimental properties. The resulting set of parameters simulates accurately the structure and atomic ordering of this material in a wide range of temperatures. However, all these models are not transferable, which means that they cannot describe mixed hybrid perovskites.

With this motivation, some of the authors of this paper developed a FF that properly describes the properties and the behavior of the compositional set CsPb(Br_*x*_I_1–*x*_)_3_ (∀*x* ∈ [0, 1]).^1^ To parametrize the mentioned FF, they developed a homemade genetic algorithm (GA). It uses the energies computed by an atomistic classical code to calculate the energies of different structures that can be compared with reference DFT results to achieve an optimal fitting of the parameters.

Following the same line of research, here we present a classical model potential, that is, a classical FF, capable of adequately describing the mixed hybrid perovskite MA_*x*_FA_1–*x*_Pb(Br_*y*_I_1–*y*_)_3_, with *x* and *y* ranging between 0 and 1. We have checked that the model reproduces the lattice parameter of each pure compound as reported in the literature. We have also produced experimental and theoretical XRD patterns to prove that the structure is also well described. After that, by running molecular dynamics simulations with the fitted FF, we have predicted some results for different combinations of ions. For example, we have obtained the diffusion coefficients of the I and Br ions at realistic conditions of pressure, temperature, and number of crystalline defects characteristic of these materials in a solar cell in operation.

The purpose of this work is 2-fold: (1) On the methodological side, we show how a combination of genetic algorithm fitting (to DFT inputs) plus human refinement of parameters can produce a fully transferable force field for mixed perovskites. (2) On the solar cells side, we report classical molecular dynamics simulations for mixed perovskites in time scales of several tenths of nanoseconds that reproduce structural and dynamical properties that are very relevant in the functioning of a perovskite solar cell.

The results obtained in this work compare very well with other theoretical and experimental data reported in the literature for crystalline perovskites. However, the results produced for the ionic diffusion coefficients cannot be directly extrapolated with the values that justify the low frequency behavior of perovskite solar cell in operating conditions (hysteresis, impedance spectra, etc.). Still, they represent the expected value for a perfectly crystalline solid with ionic halide vacancies but with no other type of defects such as grain boundaries. Considering that they were actually derived from a classical FF fitted to DFT energies, the ion diffusion coefficients here reported can be considered as the upper theoretical limit for ion dynamics in perovskite solar cells.

## The Classical Force Field

To describe the interaction of the C and N ions included in the MA and the FA and the inorganic lattice, that is, Pb, I, Br ions, and between the ions of the inorganic lattice themselves, we have used the Buckingham potential, plus the Coulomb interaction
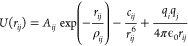
1It includes a repulsive term and an attractive term, and is usually chosen because the Pauli repulsion is well described by its exponential form. This potential model also has proven to work accurately for many materials, including other perovskites as mentioned before.^1,14,15^ In this work, based on the relative weight of the attractive term with respect to the Coulomb part, we have set the “*c*” parameter in all Buckingham interactions to zero (see the Supporting Information Figure S1 for a more detailed justification of this choice). Note that the simplification of neglecting the “*c*” parameters, in spite of its obvious validity, was not even considered in previous work, as we demonstrate in Figure S1.

To describe the nonbonded terms, which include the interaction between atoms of different organic molecules, and all the interactions including hydrogen atoms, we have employed a Lennard-Jones potential plus the Coulomb interaction
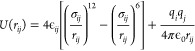
2This potential also includes a repulsive and an attractive term, in a simpler way than the Buckingham potential. It has been used in many previous works to describe this type of interactions.^17−20^

Organic molecules (Figure 1) are modeled by means of the AMBER model.^17^ This model maintains the shape of the molecule by including permanent harmonic bonds,angles and permanent dihedrals. The energy of the molecule is calculated via eq 3:
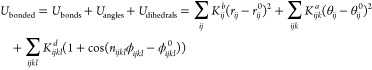
3This model has been used to describe many different organic molecules.^21^ It also allows for the possibility of distinguishing between different realizations of an atom of the same element. For instance, the parameters of the sp^3^ C of the MA can be different than the parameters of the sp^2^ C of the FA. Moreover, the parameters of the N’s depend of the number of ions with which they are bonded. Also the H’s bonded to a C or a N are also different. All these details will be further discussed in the parametrization section.

The ions in the organic molecules placed at a distance of 1 or 2 bonds (for example, the C–N in the FA and the N–N in the FA, respectively) only interact by the bonded terms. That means that a pair of bonded atoms is only determined by the bond, angle, and dihedral potentials. Ions placed at a distance of three bonds (for example a C-bonded hydrogen or a N-bonded hydrogen) feel the Lennard-Jones interaction and the Coulomb interaction, but not at its full strength. We set weighting coefficients of ^1^/_2_ for the LJ and ^5^/_6_ for the Coulomb interactions, as is typically done for the AMBER model.^17^ Ions placed at a distance of four bonds (H’s bonded to the N’s in the FA) feel the whole LJ and Coulomb interactions.

## Parameterization of the Model

### DFT Methodology

**Figure 2:**
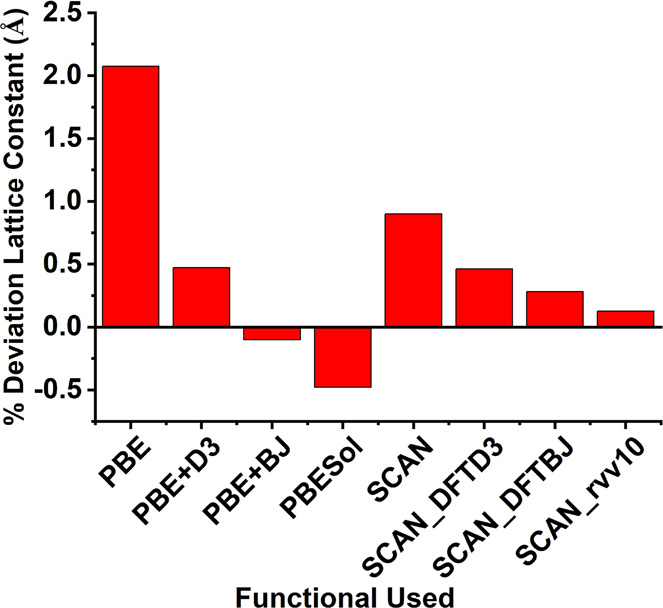
Deviations in lattice constant between theory and experiment^22^ for all functionals tested.

Our classical FF was trained against a set of reference data calculated with density functional theory (DFT) with the PBE + DFT-D3(BJ) exchange-correlation functional^23−25^ in the VASP software package.^26,27^ The choice of DFT-D3(BJ) was made based on extensive comparison of the performance of several functionals, PBE, PBEsol, and PBE with vdW corrections, such as PBE-D3,^28^ PBE-D3(BJ),^25^ and meta-GGA functional SCAN^29^ in combination with several vdW correction schemes, including D3, D3(BJ), and rvv10.^30^ Our results using pseudocubic MAPbI_3_ or FAPbI_3_ in Figure 2 indicate PBE-D3(BJ) and SCAN-rvv10 perform the best in predicting the lattice parameters and volume with minimum differences (<0.1% and <0.2% for lattice parameters and volume) compared to the experiments.^22^ This highlights the importance of including the vdW corrections when studying hybrid perovskites involving organic cations. Considering SCAN-rvv10 being computationally expensive and the need of creating a large training data set, we have chosen PBE-D3(BJ) for the rest of the DFT calculations. The outermost s, p, and d (in the case of Pb) electrons were treated as valence electrons, of which the interactions with the remaining ions were modeled by pseudopotentials generated within the projector-augmented wave (PAW).^31,32^ We have taken the initial bulk structure of cubic FAPbI_3_ and MAPbI_3_ from the literature.^33^ For all the calculations of distorted structures, the ions were relaxed while keeping the lattice parameters fixed. The energy and force convergence threshold were kept at 10^–4^ and −0.05 eV/ Å, respectively. Gaussian smearing with a sigma value of 0.02 is used. An energy cut off of 500 eV and K-points of 6 × 6 × 6 are considered for all structures of pseudocubic perovskites described below.

We have modeled electrostatic interactions using atomic point charges. The point charges of all chemical elements in the perovskite materials were calculated using the Density Derived Electrostatic and Chemical (DDEC6) method,^34,35^ which was proven to be useful in predicting the electrostatic interactions in halide perovskites.^36^

**Figure 3:**
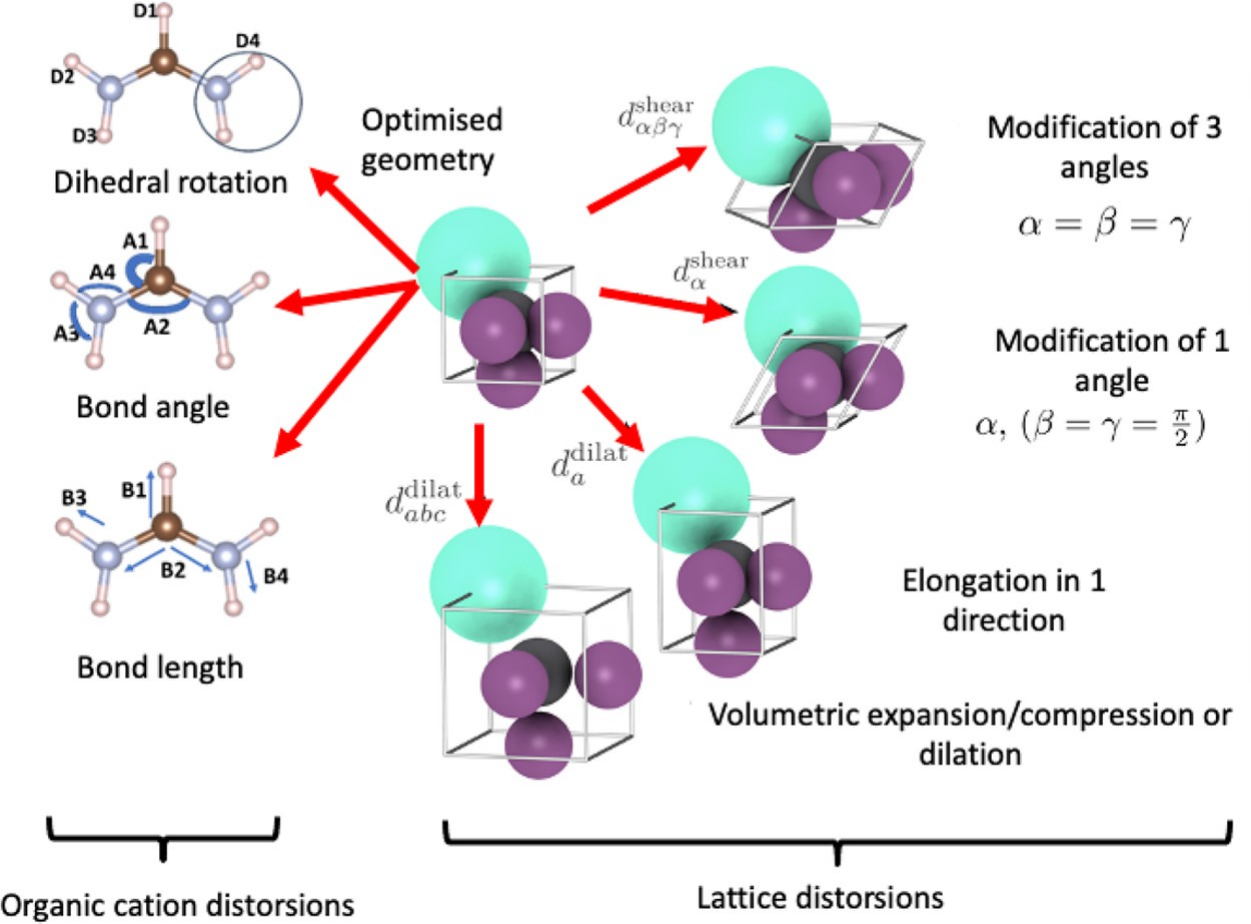
Illustration of distortions applied to calculate the DFT energies.

After optimizing the bulk structure, we have generated the distorted structures in various ways to produce a large number of distorted structures to fit the GA (see next section). The following distortions have been used (see Figure 3):^1^

- Contraction and expansion of the length of the unit cell by 0.1 Å. This was applied to all three sides all together and individual sides too while keeping two of the sides intact and varying the other one.
- Contraction and expansion of the angles of the unit cell by 1 degree. This was applied to all three angles all together and individual angles, keeping two of the angle intact and varying the other one.

Since we are dealing with perovskites which have a central organic molecule, we have also oriented them with different angles. We have rotated them along the *a*, *b*, and *c* directions by 22.5 degree individually until 360 deg to produce a set of distorted structures.

To obtain the parameters of the AMBER model, bonds, angles, and dihedrals of the organic molecule were distorted as shown in Figure 3. Further details are presented in the next subsection. To obtain the energy change produced by only distorting the organic molecule we have performed single point calculations instead of relaxations.

### Fitting Bond, Bend, and Torsion Parameters for the Organic Cations

In this section we detail how we have proceeded to obtain the force constants, angles and equilibrium distances for the interactions in the molecular mechanics of organic cations. For the bonded terms of MA we took the parameters directly from the GAFF library,^21^ because they have been proven to reproduce accurately the behavior of the molecule studied here, as have been shown by Mattoni et al.^14,15^

In the case of the FA, as shown in Figure 1, there is a double bond, which can lead to confusion when trying to reproduce its effects by a classical FF. One could think of modeling the two N’s in the FA with two different bonds, one with a single bond and the other with a double bond. However, it is more realistic to assume that both bonds are equivalent with an intermediate strength between a single and double bond (conjugated bond). Bearing all this in mind, we include six different atoms in the AMBER model as follows: C_FA_, C_MA_, N_FA_, N_MA_, H_N_, and H_C_. Added to the Pb, I, and Br, we work with nine different atoms/ions in total.

To obtain values for the FA parameters, we started by taking them from the same library. However, the parameters for the angle interaction between a “n3” nitrogen, a “c2” carbon and a “hx” hydrogen (according to the GAFF notation) are missing. That means that nobody has modeled such an interaction before, or if somebody has, it has not been included in the library. In a first try, we used angle parameters of similar elements than the ones included in this case, but the result was that the FAPbI_3_ compound was unstable when running a MD simulation with our first parameter set. To ensure good reproducibility of the experimental results and the stability of the compounds in the subsequent classical molecular dynamics simulations, we developed a set of new parameters (no GAFF based) for the intramolecular interactions of the FA cation, compatible with the AMBER formulation. We proceeded by fitting the energy of the system to energies obtained by first-principles for different distortions of the bonds, angles, and dihedrals of the FA molecule in a FAPbI_3_ unit cell. We assumed that the energy change in the unit cell is entirely due to the internal change produced in the molecule by the distortion, and not to the change in the interaction between the displaced atoms in the molecule with the elements of the crystal lattice because of the small changes in the relative distances between them. It could be argued that it would have been more efficient to work with the isolated ion using literature procedures.^37,38^ However, considering that the formamidinium is a charged molecule and that some kind of charge compensation would be needed, we found it most convenient at this stage to work with the full unit cell. Still, these alternative procedures could be more adequate for calculations involving larger organic ions.

**Figure 4:**
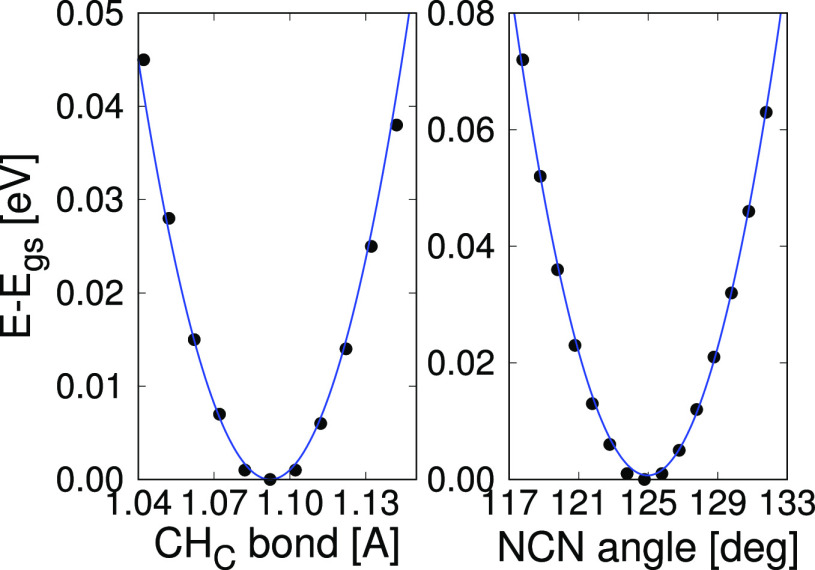
Fits to the energy difference between each distortion of the FA and the ground state, calculated by DFT. On the left we show it for the CH_C_ bond, and on the right we show it for the NCN angle, as representative cases.

The calculations were performed as follows. To parametrize the bonds, we manually generated various geometries by stretching and shrinking a chosen bond taking advantage of the symmetry of the molecule. In Figure 4 we present, as an example, the DFT energy difference (Δ*E* = *E* – *E*_gs_) for the C–H_C_ bond. In this case, a simple fit to a harmonic function give us the values of the elastic constant *K* and the equilibrium distance *r*_0_ for this bond.

To parametrize the angles is trickier, because distorting an angle to check how the energy changes causes indirectly a change in another one. For example, to obtain the parameters for the NCH_C_ angles, when increasing the left angle θ (notation of Figure 1), the right angle θ′ decreases. In this specific case, as the angles are equivalent, the elastic constants are the same. Also, the angle differences, as they are squared, will provide the same result. Thus, the energy difference can be obtained as
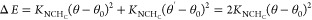
4This equation can be directly fitted to the DFT calculated reference energies. To obtain the NCN angle parameters, we distorted the molecule by increasing and decreasing the angle treating the NH_2_ group as a rigid set. As mentioned before, distorting this angle modifies indirectly the NCH_C_ angle, the parameters of which have already been calculated by eq 4 and are necessary to obtain the new ones
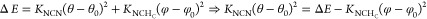
5By performing another manual fit these parameters can be calculated. This calculation is also presented in Figure 4, where we can observe that the harmonic function perfectly fits the DFT energy differences.

To obtain the parameters of the three angles that have the N as the central atom, we have performed three different sets of calculations: rotating one of the H_N_’s, the other, and both of them maintaining the angle between them. By doing that we obtain the following set of equations that allow us to determine the rest of the missing parameters
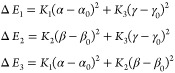
6where α, β, and γ are the bond angles (notation of Figure 1.)

To obtain the dihedral parameters, because of the complexity of the distortions and the number of possibilities, we just took some examples of distorted structures in which the dihedral contribution to the energy is clearly relevant and performed some fits to obtain a first approximation. The final values were obtained by including the first values as initial guesses in the genetic algorithm. We did that to improve the energy fitting to the DFT energy values and to avoid the complexity of the dihedral calculations.

Final values of the classical FF parameters, including Lennard-Jones, Buckingham, and AMBER models for both ion and molecules are presented in the Supporting Information.

### Electrostatic Interactions

We have put a great deal of effort into developing a classical FF which will be able to efficiently model dynamical properties of the mixed perovskites while ensuring structural stability. Besides, we tried to keep the model as simple as possible to allow for large scale atomistic simulations. For this reason, we assume here some limitations by omitting the possible polarization of the structural anions I and Br in the precise structural description. Hence, we restricted the model to the derivation of optimized atomic point charges as used in previous classical FFs for perovskite materials.^1,14,15^ For these point charges, we first developed our model using the values obtained with the DDEC6 method, as described in the DFT methodology section, and we modified them to meet the following requirements:

- The MA and FA ion molecules, as a whole should have the same charge.
- The Br and I ions should have the same charge.
- The system should be electrically neutral.
- For simplicity, charge values of the H_C_’s and the H_N_’s are fixed equal in both molecules.

First tries with the GA (*vide infra*) using the charges derived in this way did not result in a stable FF for perovskites in the molecular dynamics simulation. Inclusion of an extra parameter in the algorithm, a factor that multiplies all the charges at the same time (in order to keep charge neutrality) was demonstrated to be critical, because a small change in it would affect the energy of all the Coulomb interactions of the system, which gives them a predominant role over the rest of the parameters involved in the model. For this reason, and in order to find a set of charges that ensured physical stability of the subsequent molecular dynamics simulation with the classical FF, we modified manually the value of this common factor.

It is well-known that in hybrid metal halide perovskites,^39,40^ the frontier orbitals and the electronic band gap are mainly advanced by the Pb and I/Br orbitals rather than by the organic A cations, whose influence is only marginal. It is then reasonable to think that the interaction of the A cations with the PbI_3_ framework is less covalent and weaker^41^ and that it has a more ionic character. Within this line of thought, we found that multiplying the charges by a common factor (in particular, 2.5) such that the charges become closer to the nominal ionic charges (for instance 2 for Pb^2+^ and +1 for the whole organic cations) resulted in a stable model in the molecular dynamics simulation. The final values obtained by this “human learning” procedure are collected in Table 1. It is important to mention that the resulting high charges determine a FF in which most of the attractive contribution to the energy is due to the electric charges of ions and counterions, in line with the more ionic character of this interaction. In this respect, we refer the reader to the discussion on the choice of “*c*” parameters in the Buckingham potential, presented in the Supporting Information.

**Table 1:** Final Values of the Charges of the Classical Force Field

ion	*q*(|*e*|)	ion	*q* (|*e*|)
C_FA_	0.9717	H_*C*_	0.28
C_MA_	–0.4422	H_*N*_	0.74
N_FA_	–1.2735	Pb	1.9713
N_MA_	–0.9531	Br,I	–1.212

### Buckingham and Lennard-Jones Parameters: Genetic Algorithm

The Buckingham and Lennard-Jones parameters were obtained after fixing the values of the charges and the parameters of the AMBER model. We have used a new version of the genetic algorithm (GA) previously developed in our group,^1^ adapted to work with LAMMPS instead of GULP to calculate the energies of different structures. LAMMPS has easier compatibility with the AMBER model; moreover, it is the code that we will lately use to perform the MD simulations.

The GA is an artificial intelligence procedure that replicates natural selection. For the ground state of a compound and for its distorted structures, we have obtained the energy of each configuration by DFT calculations. These energy values will be reference data for the GA. The populations in the GA world are different sets of parameters of our classical FF. Each set of parameters will be worse or better “adapted” to reproduce the DFT energies. After each iteration, the best adapted population survives, and new children are created by mixing the genotype of the survivor sets of parameters. Random mutations are also included in the children to increase the variability of the sample, which allows exploring more regions of the configurational space. Once that a given tolerance factor is small enough or a certain number of iterations have been carried out, we obtain the final set of parameters, the one that better reproduces the DFT calculations of all those that have appeared in the GA world. More specific details of the GA can be found in ref (1). The new version of the code is available online at github.com/salrodgom/flama/releases/tag/v1.1.

Instead of running the GA for the 4 pure perovskites we want to describe, we took advantage of the possibility that the ABX_3_ formula allows directly mixing Br and I. By doing that, we are still able to perform calculations with a unit cell of five ions (including MA^+^ and FA^+^ ion molecules), and obtain the parameters of the Buckingham potential for the interaction between I and Br atoms directly from the GA. Thus, the two intermediate compounds we have studied are MAPbBr_2_I and FAPbBr_2_I. Our reference structure is always the pseudocubic one, because for pure perovskites it is the one occurring in solar cells in the operative regime. The exception is MAPbBr_2_I, which crystallizes in a tetragonal structure with a very small *c*/*a* ratio, so this approximation should be accurate enough. As we have included just one crystal phase of each compound, probably our model potential will not be able to describe phase transitions known to be occurring in these systems, (as achieved, for instance, by Mattoni et al.^15^ and Hata et al.,^14^ with their system-specific force fields). However, this is not a fundamental problem since we aim at describing the perovskite material in a solar cell in operation, where phase transitions are neither expected nor desirable. Hence, our classical FF is a “made-to-purpose” model, and it is not intended to describe the full phase behavior of the material.

We have produced and computed around 120 distorted structures for each perovskite. The included distortions can be classified in three groups: distortions involving one of the lattice parameters or the three at the same time, distortions involving one of the angles of the unit cell or the three at the same time, and rotations of the organic molecule, avoiding in all cases equivalent situations. This set of distortions gives enough DFT energy values to the GA to be able to fit the model potential with a good level of accuracy. Some of the nonisotropically distorted structures had to be discarded because the energies obtained by DFT calculations were lower than the ground state of the perfect cubic structure. This fact, which is apparently puzzling, is justified by the fact that the cubic structure is not the one with the lowest energy value of the compounds. Some distortions can bring the shape of the cell closer to the phase with the lowest energy value, and provide a lower energy value than the cubic ground state, but a higher energy than the tetragonal phase.

### Improving Transferability

After running the GA for the pure systems, and in order to have a transferable model between MA and FA, we averaged the common interactions (Pb–Pb, Pb–I, ...) obtained in the two different calculations for the two different mixed compounds. We then ran again the GA fixing the common interaction parameters values to let the other parameters readjust to the new averaged values of the common parameters.

To have a complete transferable model, we need to obtain yet the parameters of the interaction between the carbon and the nitrogen atoms of the two different organic molecules. In contrast to the case of mixing I and Br, it is not possible to run the GA with a mixture of MA and FA molecules without creating a larger unit cell, because of the stoichiometry of the formula ABX_3_. Instead, to obtain the missing parameters we averaged the known interactions between C and N atoms that have been obtained in the GA calculations using the following mixing rules
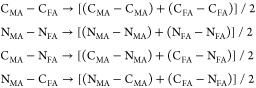


We expect this procedure to work adequately for the following reasons. The interaction between a C_MA_ and C_FA_ for example is not expected to change significantly with respect to the interaction between two C_MA_s or two C_FA_s. They are very similar ions, placed at approximately the same distance in both cases, and also surrounded by many H ions, so in the collective interaction of two whole organic molecules these will produce just a small amount of the total repulsion felt by the neighboring atom.

All parameters of the classical FF, as derived from the application of the GA and by the subsequent refining and fitting to DFT energies, can be found in the Supporting Information in Tables S1–S5.

## Force Field and Classical Molecular Dynamics Results

**Figure 5:**
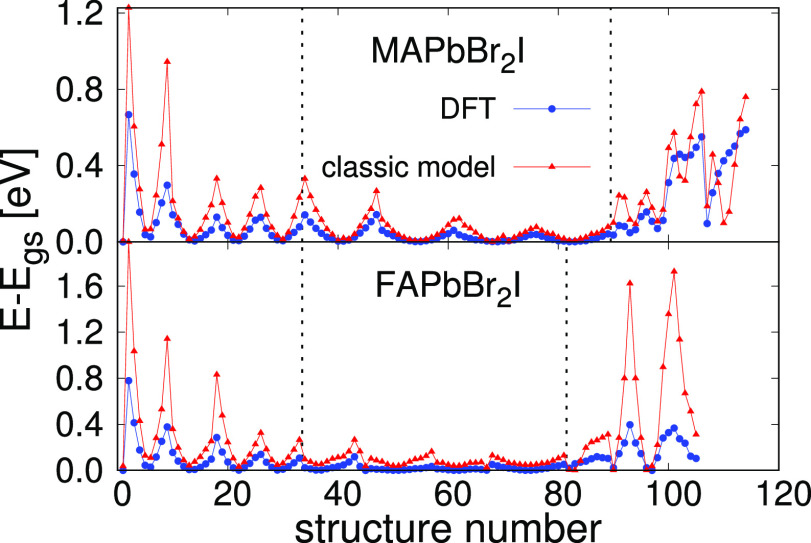
Energy difference between each distorted structure and the ground state, for the DFT calculations and for the calculations with the classical FF. We show the results for both of the compounds we have modeled employing the genetic algorithm. The dashed lines distinguish between distortions of the lattice parameter (left), distortions in angles (center), and different orientations of the organic molecule (right).

Application of the GA with the procedures described before led to a “first-guess” version of the classical FF. In Figure 5 we plot the energy of each distorted configuration with respect to the energy of the ground state, from both DFT and from the classical model. These values are important because they are the ones that appear in the function that the GA uses to establish how well a parameter set of the model reproduces the quantum results (cost function). As can be observed, both lines follow the same trend, except in the distortions corresponding to some orientations of the MA and FA cations. We attribute this anomaly to the fact that in the classical calculation there is no contribution of the rearrangement of the electronic structure upon a reorientation of the organic cations. However, in spite of these discrepancies, the behavior of the system is well reproduced, at least qualitatively.

### Simulation Details and Stability of the Model

As mentioned before, an important test when developing a FF from scratch is to confirm that the dynamics is stable and that the crystalline structure does not break down. To check this, we ran simulations with a 6 × 6 × 6 supercell, at 600 K of temperature and 1 atm of pressure for the four pure perovskites. We use a time step of 0.5 fs. Simulations were started from a perfectly cubic unit cell with a lattice parameter of 6.2 Å while forcing the system to stay in a perfect cubic cell (instead of pseudocubic) along the full span of the simulation. To obtain the volume of the system first we run a 100 ps long simulation in the NPT ensemble and averaged the volume over the last 50 ps. After that, we fixed the volume and thermalized the system in the NVT ensemble for 3 ns. We found it necessary to extend the thermalization for such a long time because of the long relaxation times of molecular orientations. A Nosé-Hoover thermostat was used to fix the temperature. Finally, we run a NVE simulation of 20 ns. The energy and the temperature plots versus time show the typical fluctuations in these kind of simulations, which means that the first-guess version of the classical FF is a model with intrinsic stability. Plots of the time evolution of the energy for these exploratory simulations can be found in the Supporting Information (Figure S2).

In spite of the encouraging result regarding stability, we observed that the lattice parameter of the MAPbI_3_ material as predicted by the simulation (6.0 Å) was too short in comparison with literature values.^14^ Although the rest of the lattice parameters were in much better agreement,^22,42^ it was found necessary to manually modify the model to correct this anomaly. Hence, we multiplied by 200 the *A* parameter of the Buckingham potential of the interactions between the C and N of the MA with the I. As this parameter represents the strength of the repulsion between these ions, increasing it leads to a larger volume of the MAPbI_3_ unit cell, as desired. It is worthwhile to compare the lattice parameters obtained here for the perovskites for which there is already a force field, that is, MAPbI_3_ and MAPbBr_3_. The reported results are 6.25 and 5.9 Å, respectively.^14,15^

### Lattice Parameters

**Figure 6:**
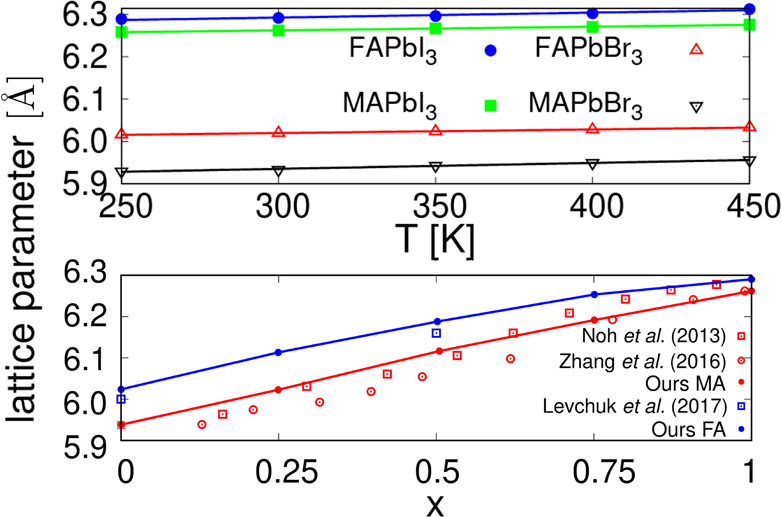
Lattice parameters of the four pure compounds we can model, obtained using the final version of the classical FF. We represent them as a function of the temperature (top) and as a function of the I/Br ratio (bottom). Red and blue symbols correspond to MA and FA perovskites, respectively. For the sake of comparison, we have included experimental data points taken from the literature.

The parameters of the final version of the classical FF are presented in the Supporting Information. Lattice parameters, as predicted by the classical FF, are shown in Figure 6 as a function of temperature and composition. Our simulated data compare very well with the experimental results of Noh et al.,^43^ Zhang et al.,^44^ and Levchuck et al.^45^

We observe that the FF replicates the expected behavior, with the FA perovskites larger than MA ones, and with a systematic expansion of the lattice with respect to temperature and increasing concentration of iodine atoms. In this respect, we calculated the thermal expansion coefficients using the expression
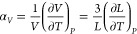
7

Results can be found in Table 2. All of them are of the order of 10^–5^ K^–1^ which compares well with an α_*V*_ value of 1.57 × 10^–4^ K^–1^ for the MAPI^46^ and α_*V*_ = 2.2 × 10^–4^ K^–1^ for the mixture FA_0.5_MA_0.5_PbI_3_.^47^ However, in the first reference, the authors indicate that their value is fairly high, and that other thin-film solar cell materials present an α_*V*_ of the order of 10^–5^ K^–1^, which would be in better agreement with our results.

**Table 2:** Volumetric Thermal Expansion Coefficients of the Four Pure Compounds, Calculated by eq 7

compound	α_*V*_ (K^–1^)
FAPbI_3_	5.5 × 10^–5^
MAPbI_3_	4.1 × 10^–5^
FAPbBr_3_	4.2 × 10^–5^
MAPbBr_3_	7.0 × 10^–5^

### XRD Patterns

**Figure 7:**
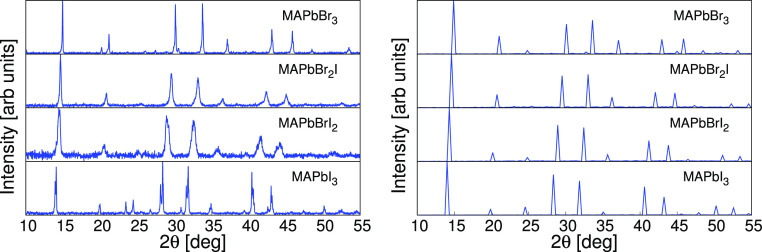
Experimental (left) and simulated (right) XRD patterns of the MA perovskites. Experimental data correspond to the cubic phase except MAPbI_3_. See SI for details.

**Figure 8:**
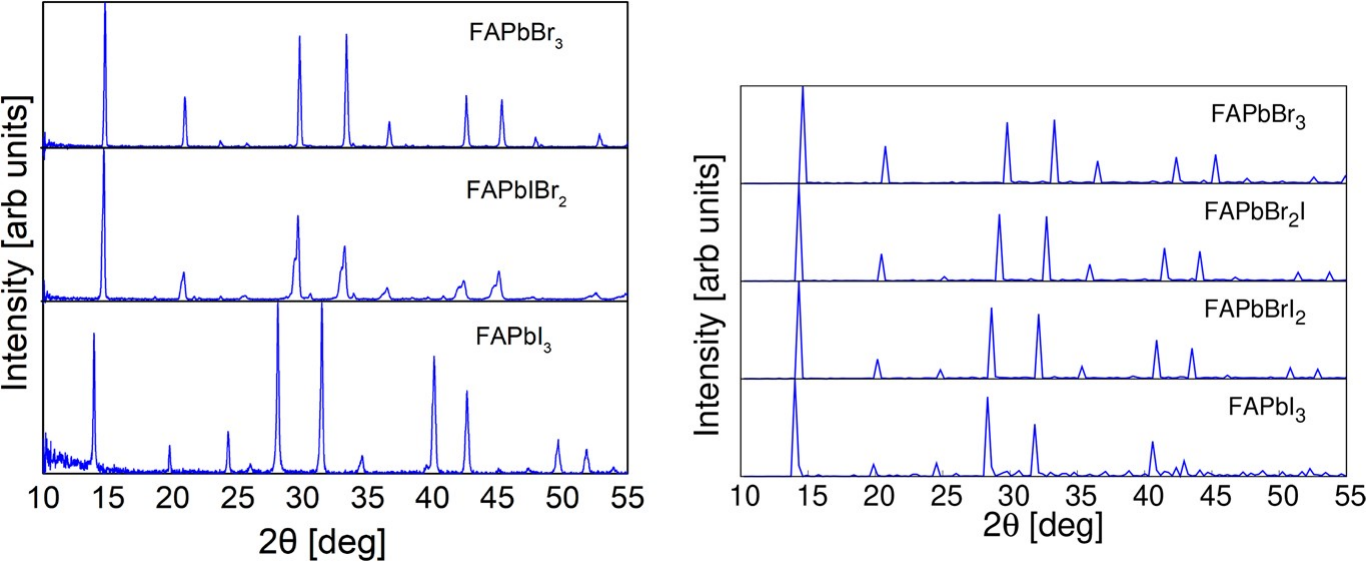
Experimental (left) and simulated (right) XRD patterns of the FA perovskites. Experimental data correspond to the cubic phase. See SI for details.

We have checked how well the classical FF reproduces the experimental XRD patterns of the studied perovskites. Simulated and experimental results are compared in Figure 7 for MA perovskites and in Figure 8 for FA perovskites. Experimental details about the preparation and XRD characterization of perovskite films and powders are collected in the Supporting Information.

The substitution of the iodide ion for the smaller bromide ion in both FA and MA perovskite systems is followed by a shift toward higher angles in the positions of the XRD pattern peaks because of the corresponding reduction in interplanar distances. This shift is accurately reproduced by CMD simulations with the GA-fitted classical FF. In general, XRD patterns are well replicated by the model with a cubic phase, in spite of the fact that in the experiment the perovskite is in a tetragonal phase with small *c*/*a* ratio. However, the similar shift produced by FA/MA substitution in the α-black phase of FA perovskites is not completely caught by the model although this deviation is actually very small (see Supporting Information, Figure S3).

## Dynamical Properties

One of the main objectives of our made-to-purpose model is to reproduce correctly the dynamical properties and the ion migration features of the mixed perovskites at typical working conditions of the solar cell. Specifically, we have run simulations of 10 ns in the same conditions we used to check the stability of the model to obtain the diffusion coefficients of the different ions. To achieve that, we have calculated the mean squared displacement (MSD) of each one of them. MSD is defined as a statistical average of the change in the ion positions in the simulation
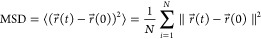
8In this equation, the index *i* runs over all the *N* migrating ions. If the ions follow a diffusive regime, the MSD would increase linearly with time, and will be related with the diffusion coefficient as
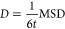
9

Results for typical MSDs can be found in the Supporting Information in Figure S4. Within this formalism, we have not been able to appreciate diffusion of neither Pb ions nor the organic molecules, even using a large number of vacancies and a temperature of 600 K. This is sort of expected knowing the activation energies of the Pb and the MA (2.31 and 0.84 eV, respectively) and also the reported diffusion coefficient of the MA (10^–16^ cm^2^ s^–1^).^48^ However, we succeeded in obtaining the diffusion coefficients of the halide ions. For each pure compound, we ran simulations for five temperature values and for two alternative numbers of vacancies. Moreover, for each combination of these two variables, we run three simulations with different initial conditions, locating the vacancies in different positions, to improve the statistics and reduce the numerical error.

**Figure 9:**
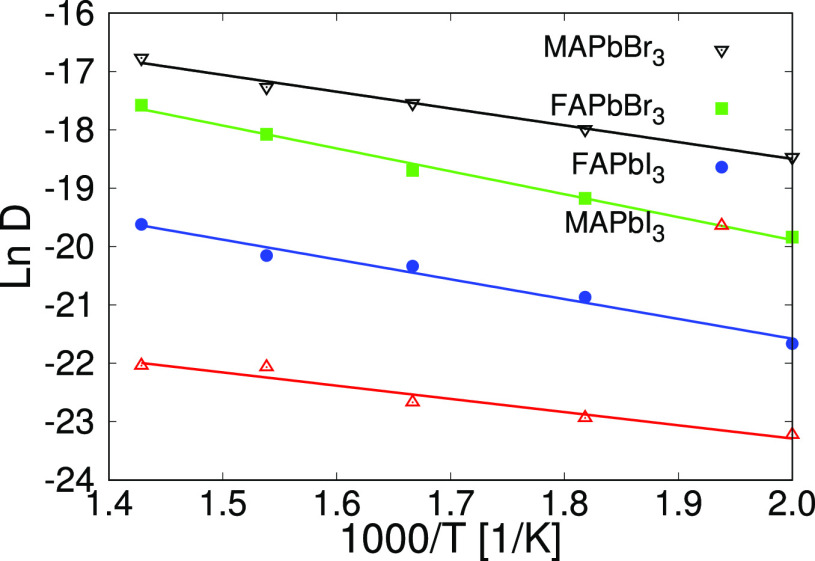
Fit to the Arrhenius equation (eq 10) for the four pure perovskites in simulations with 24 vacancies. Each point in this plot is the average of the results obtained in three simulations with different initial conditions.

It is important to mention that at 300 K diffusion is so slow that the required simulation time exceeds common computational resources. So we decided to calculate the diffusion coefficient at higher temperatures and extrapolate it to room temperature using the Arrhenius equation
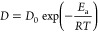
10where *D*_0_ is a temperature-independent preexponential factor, *E*_a_ is the activation energy for diffusion, and *R* is the gas constant. In Figure 9 we plot the fit to eq 10 for the four pure perovskites, from simulations run with 24 vacancies (approximately 5 × 10^20^ cm^–3^). Each point is an average over the results of three different initial conditions. It can be seen that Arrhenius equation fits accurately the simulated data. From the fitting formula we can extrapolate the diffusion coefficients at 300 K and calculate the activation energy of each species. The results are presented in Table 3.

**Table 3:** Diffusion Coefficient at 300 K and Activation Energy for Each Pure Perovskite with Two Concentrations of Vacancies in the Supercell

compound	*N* vac	*D*_300K_ [cm^2^ s^–1^]	*E*_a_ [eV/ion]
MAPbBr_3_	12	1.4 × 10^–10^	0.27 ± 0.02
	24	5.2 × 10^–10^	0.25 ± 0.02
FAPbBr_3_	12	1.6 × 10^–11^	0.32 ± 0.02
	24	4.6 × 10^–11^	0.34 ± 0.02
MAPbI_3_	12	-	-
	24	8.0 × 10^–12^	0.20 ± 0.03
FAPbI_3_	12	5.0 × 10^–12^	0.29 ± 0.02
	24	1.4 × 10^–11^	0.29 ± 0.02

We find values of the order of 10^–10^–10^–11^ cm^2^/s for the diffusion coefficients and activation energies between 0.2 and 0.33 eV. We also observe that values from the simulations with 24 vacancies are more or less twice the ones of the simulations with 12 vacancies, as predicted by the theory (*vide infra*). Moreover, the activation energies of the studied systems do not depend on the number of vacancies used the simulation. Activation energy values are in the order of those reported in the literature, both in experiments and DFT calculations: 0.17 eV,^49,50^ 0.10 eV,^15^0.1 eV,^51^ 0.58 eV,^48^ and 0.22–0.28 eV.^52^ For MAPbI_3_ the diffusion is so slow that no diffusion coefficient could be derived from a simulation with 12 vacancies of just 10 ns.

From the results obtained with two concentrations of vacancies, we can infer the jumping rate, which is independent of the vacancy concentration, and use it to extrapolate the diffusion coefficient to a realistic density of vacancies at room temperature. To do so, we have applied the following procedure, described in the references.^53,54^ The diffusion coefficient *D*_*i*_ of the specie *i* (which for us will always be I or Br) is related to the jumping rate of the same species Γ_*i*_ by
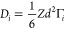
11where *Z* is the number of neighbors (eight in our case) and *d* is the jumping distance. In our case, as we are considering cubic cells and jumps from one site of the octahedra to another, we have
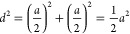
12where *a* is the lattice parameter. From eq 11 we calculate the jumping rate, that is also related to the jumping rate of the vacancies Γ_*v*_ of that species by
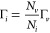
13where the subindex *v* is used for vacancies, and *N*_*v*_ and *N*_*i*_ are the vacancies and ions concentrations, respectively. In fact, as concentrations always appear related in a fraction, we can directly use the total number of vacancies and ions, which is easier because we fix these values when creating the supercell of the simulation. Once that we know Γ_*v*_, we can calculate the diffusion coefficient at 300 K with the number of vacancies expected at room temperature via
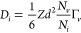
14

We have calculated the expected density of vacancies taking into account the reaction enthalpies reported in ref (55). In that work, authors present three different reactions in which one or more I vacancies in the MAPbI_3_ perovskite are involved, which is the defect that enables diffusion. We expect these reactions to provide a more realistic vacancy concentration than other calculations that use DFT to obtain the energy formation of a defect in isolation.^56^ Taking into account the three reactions studied in ref (55), 4.25% of the I sites would be empty, which corresponds to a vacancy concentration of the order of 10^20^ cm^–3^ for MAPbI_3_. In any case, our procedure makes it possible to estimate the actual diffusion coefficient for any temperature and vacancy concentration, should this be required for different experimental conditions or alternative arguments by which a different vacancy concentration can be argued.^57,58^

As the volume of the unit cell of all the studied perovskites is different, we find it more sensible to extrapolate the result of 4.25% empty halide locations rather than the vacancy concentration to estimate this parameter in all perovskites. Hence, using eq 14 we are in the position to estimate the diffusion coefficient of the halides in the four pure perovskites at a temperature of 300 K for a realistic vacancy concentration. The results are presented in Table 4.

**Table 4:** Predicted Values of the Diffusion Coefficient of Halides in the Four Pure Compounds with Pseudocubic Structure at 300 K and a Vacancy Concentration Corresponding to 4.25% of Empty Halide Sites (Approx. 10^20^cm^–3^)

perovskite	*D*_300K_ [cm^2^ s^–1^]
MAPbBr_3_	4.6 × 10^–10^
FAPbBr_3_	4.5 × 10^–11^
MAPbI_3_	9.2 × 10^–12^
FAPbI_3_	1.4 × 10^–11^

As inferred from the results, we predict a faster diffusion for the Br ions than for the I. This is an important result because it would be a factor for intrinsic instability and phase segregation of perovskites in operation.^59,60^ On the other hand, the impact of Br/I substitution on the diffusion coefficient is much more acute in the FA perovskites than in the MA ones. Since the molecular size of the FA cation is larger (4 versus 3 Å), we attribute this observation to a steric effect. This is also noticed in the generally larger activation energies obtained for the FA perovskites (see Table 3).

Diffusion coefficient values obtained for the estimated vacancy concentration at room temperature are below those reported by other simulation works.^54,61^ However, as discussed before, they were obtained for a much larger concentration of vacancies. Interestingly, our estimations a very similar to those inferred by Kennard et al.^62^ from phase separation experiments: 10^–11^ cm^2^ s^–1^. Our results are also 1 order of magnitude higher than some other results reported in the literature.^48,63^ For instance, in order to reproduce the hysteresis of the IV curve for typical scan rates of 100 mV/s and a (thermally activated) frequency peak of 0.1–1 Hz in the impedance spectra, a diffusion coefficient of the order of 10^–12^ cm^2^/s is required for MAPbI_3_ cells.^63^ However, we must bear in mind that the values obtained with the procedure we devised here correspond actually to a perfectly cubic crystalline solid, with halide vacancies but without other defects such as grain boundaries. This other type of defect, commonly present in solution processed perovskites, would significantly delay the ion dynamics in solar cells.^64^ The values here reported would then correspond to the theoretical upper limit for ion diffusion in perovskite crystals, as predicted using exclusively first-principles arguments.

We have also run simulations for the mixtures presented in Table 5. In this case, the linearity predicted by eq 14 with respect to the concentration of vacancies is also approximately maintained, and the activation energies are of the same order as for the pure compounds. For perovskites that include I and Br ions, we have computed a combined diffusion coefficient for both of them. We observe that the diffusion increases with increasing values of the Br/I ratio. For the mixed MA-FA case with, we predict an easier mobility for the Br ions when they are in a MA environment with respect to a FA one. We would expect the same behavior when working with I instead of Br.

**Table 5:** Predicted Diffusion Coefficients for Various Combinations of the FA Compounds and the MA-FA Mixture

Compound	*N* vac	*D*_300K_ [cm^2^ s^–1^]	*E*_a_ [eV/ion]
FAPbBr_1_I_2_	12	1.0 × 10^–11^	0.27
	24	1.4 × 10^–11^	0.31
FAPbBr_2_I_1_	12	1.1 × 10^–11^	0.32
	24	2.1 × 10^–11^	0.33
MA_0.5_FA_0.5_PbBr_3_	12	3.6 × 10^–11^	0.29
	24	6.2 × 10^–11^	0.32

**Figure 10:**
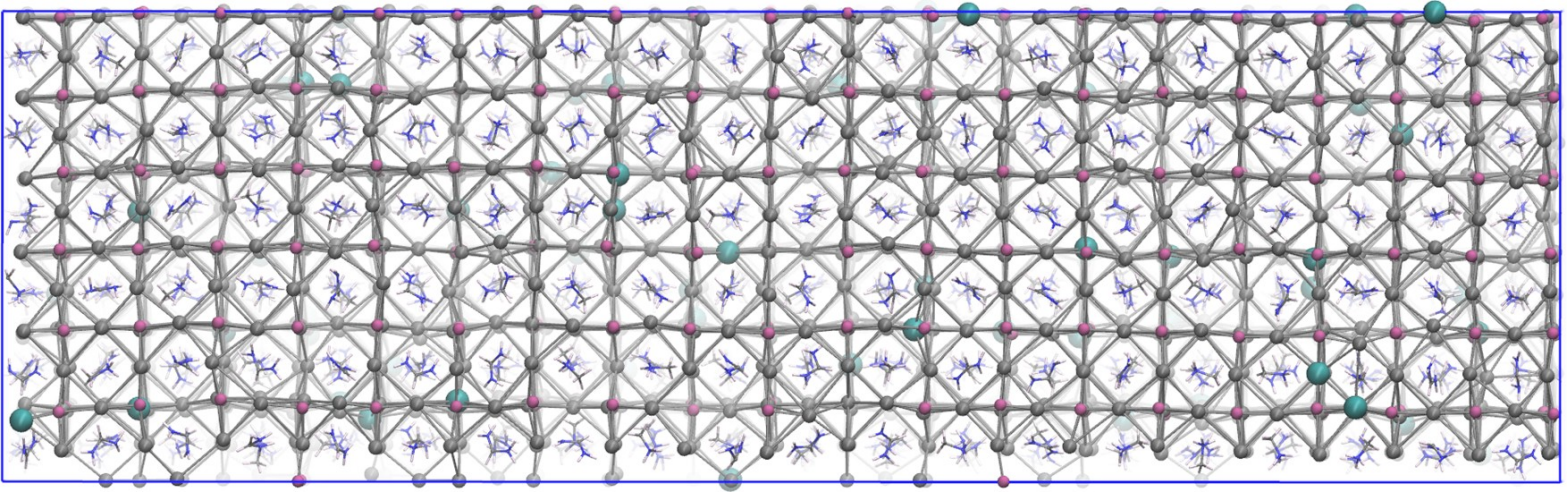
Snapshot of the 6 × 6 × 20 structure. Code color of Pb, I, Br, C, N, H atoms: mauve, silver, seawater (larger spheres), gray, blue, and white, respectively.

In order to rule out system size effects, and to get closer to the macroscopic limit, we have extended the simulations to a 6 × 6 × 20 supercell (∼8600 atoms) and a total simulation time of 40 ns. These longer simulations were run on a composition of MA_0.29_FA_0.71_PbBr_0.1_I_2.9_^65^ and with 4.25% empty halide sites (see Figure 10). In these simulations an external electric field up to 0.01 V Å^–1^ along the *z* direction has been applied. Ionic segregation was not observed to occur in the system, probably because it is a process that takes place in longer time scales.^63^ However, we have confirmed that larger samples produced statistically equal values of the ion diffusion coefficients than in the simulations with smaller samples, shorter simulations, and without electric field, showing that the electric field does not affect the ion migration properties at least in the short time scale.

## Conclusions

We have derived a classical force field with transferable properties suitable to describe mixed perovskites (MA_*x*_FA_1–*x*_Pb(Br_*y*_I_1–*y*_)_3_ ∀*x*, *y* ∈ [0, 1]) by molecular dynamics with up to 8600 atoms and simulation times of up to 40 ns. The force field parameters were determined by means of a genetic algorithm that seeks the best set that fits DFT energies for a combination of crystalline structures with several degrees of distortion with respect to the equilibrium configuration, and then refined to ensure stability of the molecular dynamics simulations. The classical model reproduces correctly the crystalline structure (as inferred from the comparison of the simulated XRD patterns with experimental ones), the expansion of the structure upon Br/I substitution, and the thermal expansion coefficients. It also predicts activation energies for halide diffusion in agreement with literature values. Using theoretical arguments based on jumping rates and an extrapolation scheme based on the Arrhenius equation, we have been able to predict the ion diffusion coefficients of mixed perovskites at room temperature and for realistic concentrations of ion vacancies. Being based on long simulations and big samples for a DFT-fitted classical forced field, these values can be considered as optimal values (within the accuracy of the procedure) obtainable from first-principles for perfectly crystalline solids with no defects other than halide vacancies. As real perovskites synthesized for solar cell operation do always have a variable amount of defects and grain boundaries, the value predicted in this work can be considered as a theoretical upper limit for ion diffusion in real perovskites for photovoltaic applications.

In spite of the general good performance of the force field, we must note that our method is not fully automated and requires human refinement at some stages, that is, rescaling of charges and the repulsive term in the Buckingham potential to fit experiments. In addition, it is important to clarify that the reported parameter set is not aimed either at describing phase transitions or perovskites which are not in a cubic or pseudocubic structure. This would require additional training with an expanded set of DFT energies. Still, our method is very versatile and, hence, it could be considered a starting point in the development of transferable force fields in these and other perovskites.

## Supporting Information Available

Classical force field parameters and additional XRD and simulation data; analysis of “*c*” parameters in Buckingham potential; stability of the classical Molecular Dynamics simulations; experimental details of the synthesis of perovskites; XRD data as a function of the MA/FA ratio; mean square displacements for ionic migration from the simulations (PDF)

## Notes

Data and software availability. The VASP software version 5.4.1 to generate the reference DFT data can be purchased at the https://www.softwaresuggest.com/us/vasp Web site. An evaluation or demo license of this software package is provided by the vendor upon request. The LAMMPS code is freeware and can be downloaded free of charge from https://www.lammps.org. Input files needed to reproduce the DFT and CMD simulations shown in this paper can be downloaded from https://github.com/salrodgom/PerovskiteMAFAPbBrI3. The genetic algorithm software FLAMA is available in the public repository: https://github.com/salrodgom/flama/releases/tag/v1.1 All the figures were made with GNUPLOT 5.2 which is freely available from the http://www.gnuplot.info/download.html Web site.
