# Supplementary material for: Transferable Classical Force Field for Pure and Mixed Metal Halide Perovskites Parameterized from First-Principles
Source: J Chem Inf Model. 2022 May 16;62(24):6423–35. doi: 10.1021/acs.jcim.1c01506 (PMC9795557; doi:10.1021/acs.jcim.1c01506)
Supplement: Supplementary file 1 — ci1c01506_si_001.pdf [file ci1c01506_si_001.pdf]

Supporting Information

Transferable Classical Force Field for Pure and Mixed Metal Halide Perovskites Parameterized from First Principles

Juan Antonio Seijas-Bellido,† Bipasa Samanta,‡ Karen Valadez-Villalobos,† Juan Jesús Gallardo,¶ Javier Navas,¶ Salvador R. G. Balestra,†,§ Rafael María Madero Castro,† José Manuel Vicent-Luna,‖ Shuxia Tao,‖ Maytal Caspary Toroker,‡ and Juan Antonio Anta∗,†

†Área de Química Física, Universidad Pablo de Olavide, 41013 Seville, Spain

‡Department of Materials Science and Engineering, Technion–Israel Institute of Technology, Haifa 3200003, Israel

¶Departamento de Química Física, Facultad de Ciencias, Universidad de Cádiz, E-11510 Puerto Real, Cádiz, Spain

§Instituto de Ciencia de Materiales de Madrid, Consejo Superior de Investigaciones Científicas (ICMM-CSIC) c/Sor Juana Inés de la Cruz 3, Madrid 28049, Spain

‖Materials Simulation and Modelling, Department of Applied Physics, Eindhoven University of Technology, P.O. Box 513, 5600MB, Eindhoven, The Netherlands

E-mail: anta@upo.es

1. Classical Force field parameters

As discussed in the main text, a Buckingham potential is used to model the interactions between inorganic components in the perovskite material, i.e. Pb, I and Br, and between the inorganic components and the organic molecules. Intermolecular interactions between the organic molecules themselves are treated via Lennard-Jones potentials. Intramolecular interactions in the organic molecules are modeled via the AMBER model. All electrostatic interactions are calculated using Coulomb potentials with point charges.

All parameters of the force field, as derived from the application of the GA and by manual fitting to DFT energies can be found in Tables S1, S2, S3, S4 and S5

Table S1. Atomic parameters and labels used in Tables S2 and S3. “MA” and “FA” refer to methylammonium and formamidinium, respectively.

Atom	Label	Mass (dalton)	Charge (|e|)
C(FA)	1	12.0107	0.9717
C(MA)	2	12.0107	-0.4422
N(FA)	3	14.0067	-1.2735
N(MA)	4	14.0067	-0.9535
H(C)	5	1.00784	0.28
H(N)	6	1.00784	0.74
Pb	7	207.1999969	1.9713
I	8	126.9044724	-1.212
Br	9	79.904	-1.212

Table S2. *Intermolecular* force field parameters.

Atomic pair		Interaction type	A / ε (eV)	ρ / σ (Å)
1	1	Lennard-Jones	7.26×10^{-3}	2.94
1	2	Lennard-Jones	0.005883	2.87
1	3	Lennard-Jones	3.46×10^{-3}	3.74
1	4	Lennard-Jones	0.0027	3.53
1	5	Lennard-Jones	8.33×10^{-3}	3.52
1	6	Lennard-Jones	8.28×10^{-6}	3.32
1	7	Buckingham	4.57×10^{6}	0.133
1	8	Buckingham	3.60×10^{6}	0.206
1	9	Buckingham	2.01×10^{5}	0.215
2	2	Lennard-Jones	4.50×10^{-3}	2.80
2	3	Lennard-Jones	0.00272	3.53
2	4	Lennard-Jones	1.99×10^{-3}	3.32
2	5	Lennard-Jones	6.95×10^{-3}	1.29
2	6	Lennard-Jones	6.28×10^{-3}	2.48
2	7	Buckingham	$3.63 \times 10^{+6}$	0.150
2	8	Buckingham	$1.95 \times 10^{+7}$	0.127
2	9	Buckingham	$4.25 \times 10^{+6}$	0.148
3	3	Lennard-Jones	4.78×10^{-3}	1.62
3	4	Lennard-Jones	0.00331	2.704

3	5	Lennard-Jones	8.92×10^{-3}	2.15
3	6	Lennard-Jones	5.90×10^{-3}	2.88
3	7	Buckingham	1.62×10^{5}	0.274
3	8	Buckingham	4.41×10^{6}	0.210
3	9	Buckingham	4.22×10^{6}	0.194
4	4	Lennard-Jones	1.85×10^{-3}	3.79
4	5	Lennard-Jones	5.38×10^{-4}	2.09
4	6	Lennard-Jones	6.29×10^{-4}	3.67
4	7	Buckingham	3.14×10^{3}	0.174
4	8	Buckingham	6.87×10^{8}	0.182
4	9	Buckingham	$7.56\ 10^{5}$	0.227
5	5	Lennard-Jones	0.00412	1.54
5	6	Lennard-Jones	0.00235	2.07
5	7	Lennard-Jones	0.00115	3.675
5	8	Lennard-Jones	0.00615	2.624
5	9	Lennard-Jones	0.00325	2.920
6	6	Lennard-Jones	0.00151	2.042
6	7	Lennard-Jones	0.00350	2.050
6	8	Lennard-Jones	0.006330	1.284
6	9	Lennard-Jones	0.002468	1.405
7	7	Buckingham	2.751×10^{5}	0.3788
7	8	Buckingham	4.874×10^{5}	0.2195
7	9	Buckingham	3.592×10^{5}	0.2169
8	8	Buckingham	2.574×10^{6}	0.1093
8	9	Buckingham	2.294×10^{6}	0.2167
9	9	Buckingham	5.885×10^{5}	0.1582

Table S3. *Intramolecular* AMBER bond parameters for organic molecules (see Eq. 3 in main text)

**Atomic pair**		***k*** **(eV)**	r^0 **(Å)**
1	3	64.1318	1.3109
1	6	16.5373	1.0922
3	6	20.4670	1.0201
2	4	25.4627536	1.499
2	5	29.3740962	1.091
4	6	32.0018940	1.033

Table S4. *Intramolecular* AMBER angle parameters for organic molecules (see Eq. 3 in main text)

**Atomic triad**	***k*** **(eV)**	θ^0 **(degrees)**
N C N (FA)	2.5600	124.7

N C H (FA)	1.8682	117.6
H N C (up Hs)(FA)	1.6852	116.3
H NC (down Hs)(FA)	1.6204	124.9
HNH (FA)	1.5198	118.7
Hc C N (MA)	4.2495	107.9
C N H (MA)	4.0067	110.1
Hc C Hc (MA)	3.3823	110.7
H N H (MA)	3.5124	108.1

Table S5. *Intramolecular* AMBER dihedral parameters for organic molecules (see Eq. 3 in main text)

**Atomic dihedral**	***k* (eV)**	***n***	**ϕ^0 (degrees)**	**Weighting factor**
HNCN (FA)	0.0755	2	180	0
HNCHc (FA)	0.1450	2	180	0
MA	0.0067473	3	0	0

2. Analysis of "c" parameters in Buckingham potential

There is an important aspect of the model that needs to be mentioned. When running the GA, the "c" parameters of the Buckingham potential of some pairs of ions fluctuated very strongly between zero and very high values. To check if these changes would be relevant for our model we took extreme "c" values coming from the GA calculation and evaluated the expression

$$\frac{\text{Buck}}{\text{Buck}(c=0)} = \frac{A_{ij}\exp\left(-\frac{r_{ij}}{\rho_{ij}}\right) - \frac{c_{ij}}{r_{ij}^6} + \frac{q_i q_j}{4\pi\epsilon_0 r_{ij}}}{A_{ij}\exp\left(-\frac{r_{ij}}{\rho_{ij}}\right) + \frac{q_i q_j}{4\pi\epsilon_0 r_{ij}}}. \qquad \text{(S1)}$$

For further assurance, we also did the same with the "c" values of references [1,2] and plot the same equation. The result is presented in Fig. S1

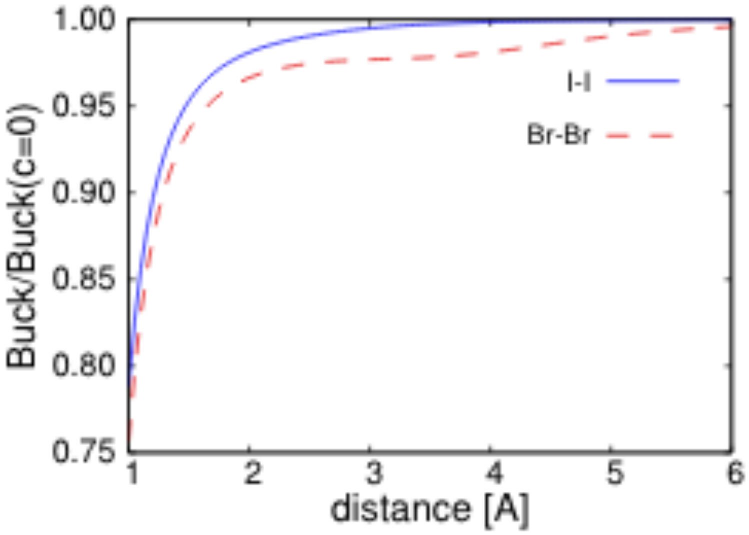

Figure S1. Plot of Eq.(S1) for the published Br-Br and I-I interactions in references [1,2]

The average distance between Br or I ions in our system is approximately 4.3Å. It can be observed in the plot that between 3 and 6 Å the "c" values modify the Buckingham interaction by 3% at the most, so it seems reasonable to neglect this parameter in the fitting and reduce the number of adjustable parameters. In our case, the "c" parameters were also not affecting the energies of the system appreciably, so any random and extreme mutation in the "c" parameter along the GA calculation could survive if the other parameters of its same phenotype improved the cost function of the GA.

It could be argued that two halides could get close enough so that the "c" parameter will become relevant. To rule out that possibility we ran simulations long enough to allow for diffusion of ions beyond the ballistic regime. Simulations with various realizations of the "c" parameter produced equivalent values of the diffusion coefficient (within the numerical error of the simulation). This is sort of expected, because when an halide ion moves to the position of a vacancy (which is the only possible diffusion mechanism in the time scale covered in this work), it follows a trajectory far apart from any other ion, as shown in Ref.[3] Based on these exploratory simulations, we chose then to set all the "c" parameters equal to zero and reduce the total number of free parameters in the GA, hence reducing the complexity of the FF and the procedure without losing accuracy.

3. Stability in the Classical Molecular Dynamics Simulations

The physical suitability of the force field parameters are checked by confirming that there is no energy drift in the CMD simulations. In Figure S2 the results of a dynamics of 20 ns is shown. No significant energy drift is observed, being the mean values of the energy fairly constant in that time interval.

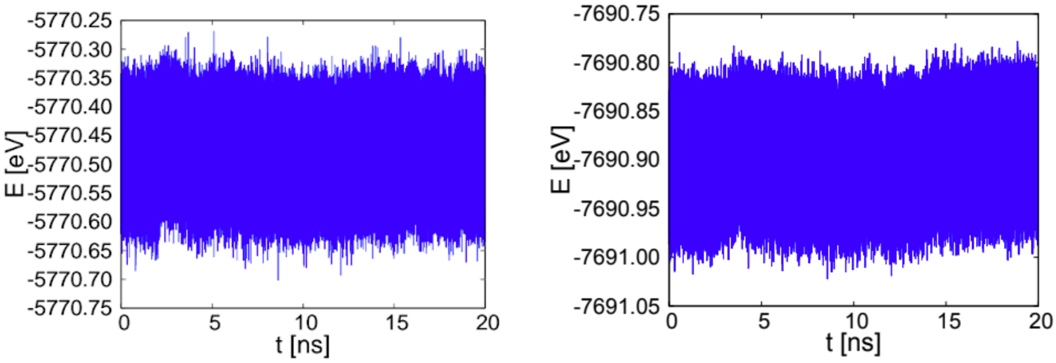

Figure S2. Energy versus time for MAPbBr and FAPbI simulations

4. Synthesis method $MAPb(I_{(1-x)}Br_x)_3$ (x = 0; 0.33; 0.67; 1) samples.

We have prepared our own perovskite materials for comparison with the simulations. The methyl ammonium halides used in the preparation of these samples were synthesized in our labs while the rest of solvents (PbI_2, $PbBr_2$ and γ-butyrolactone) were provided by Sigma Aldrich.

Table S6. Molar fraction of bromide in the prepared samples (XRD in Figure 8, main text)

Sample	x (bromide content)
$MAPbI_3$	0
$MAPbI_2Br$	0.33
$MAPbIBr_2$	0.67
$MAPbBr_3$	1

Solutions with 1 M concentration, in γ-butyrolactone, were prepared varying the composition of the halides (I^- and Br^-) as shown in Table S7. The resulting solutions were kept at 60° C under

stirring until a dry solid was obtained. The solid samples were kept hermetically sealed and protected from light and humidity to avoid degradation before characterization in the X-ray diffractometer (XRD). The XRD patterns shown in figure 8, in the main text, agree with the expected results for cubic phases for the samples with Br = 0.33. 0.68 and 1, while the characteristic peaks at around 25 degrees obtained for the $MAPbI_3$ sample correspond to the tetragonal phase.

The XRD patterns were obtained a using a Bruker® D8 Advance A25 diffractometer emitting Cu-Kα radiation (1.540 Å) and a Lynxeye detector. The measurement range was from 3° to 75°in the 2θ range, with an accuracy of 0.020°. The measurement conditions were 40.0 kV and 40.0 mA.

5. Synthesis method of $FA_{(1-y)}MA_{(y)}PbX_3$(X= I, Br) powders and films

$FA_{(1-y)}MA_{(y)}PbX_3$(X= I, Br) perovskite films. The perovskite films were deposited on FTO substrates with a mesoporous TiO_2 layer. The m-TiO_2 layer was deposited by spin coating a commercial TiO_2 paste (transparent TiO_2 paste, GreatCell Solar) diluted in ethanol (1:5 weight ratio) at 2000 rpm for 10 seconds. The film was then heated at 500 °C for 30 minutes.

The perovskite precursor solutions were prepared by mixing the precursor amounts specified in table B for each film stoichiometric composition in N-dymethylformamide (DMF, Acros organics)/dimethyl sulfoxide(DMSO, Acros organics)(1:0.95 v/v). This solution was further dissolved in a mixture of DMF/DMSO with a ratio tuned according to the relative humidity in the laboratory environment.

Table S7. Amounts of PbI_2 (Sigma Aldrich, 99%), $PbBr_2$ (Sigma Aldrich, $\geq$98%), methylammonium iodide (MAI, GreatCell Solar) and formamidinium iodide (FAI, GreatCell Solar) used to produce the characterized films and powders.

	**PbI_2**	**$PbBr_2$**	**MAI**	**FAI**
**$FAPbI_3$**	1.5 mM	-	-	1.5 mM
**$FA_{(0.5)}MA_{(0.5)}PbI_3$**	1.5 mM	-	0.75 mM	0.75 mM
**$FA_{(0.3)}MA_{(0.7)}PbI_3$**	1.5 mM	-	1.05 mM	0.45 mM
**$FAPbI_2Br$**	1.0 mM	0.5 mM	-	1.5 mM
**$FA_{(0.5)}MA_{(0.5)}PbI_2Br$**	1.0 mM	0.5 mM	0.75 mM	0.75 mM
**$FAPbIBr_2$**	0.5 M	1.0 mM	-	1.5 mM
**$FA_{(0.5)}MA_{(0.5)}PbIBr_2$**	0.5 M	1.0 mM	0.75 mM	0.75 mM
**$FAPbBr_3$**	-	1.5 mM	-	1.5 mM

The perovskite films were deposited on the FTO/m-TiO_2 substrates, in ambient air, by spin coating at 4500 rpm for 50 s. DMF was selectively washed with diethyl ether at the second 3 of the spin coating cycle. The films were subsequently heated at 150 °C for 30 minutes.

$FA_{(1-y)}MA_{(y)}PbX_3$(X= I, Br) perovskite powders. The perovskite powders were prepared by mixing the same amounts found in table B for the three reported powder compositions. The

precursors were dissolved in 1 mL ofγ-butyrolactone and stirred at 60°C overnight. The resulting solid was washed with diethyl ether three times and dried at 60° in an oven for 16 hours.

The XRD characterization was performed in a Bruker diffractometer using Cu Kα radiation, with a step size of 0.02 °2θ and 0.2 s per step. The XRD characterization was performed in a Bruker diffractometer using Cu Kα radiation, with a step size of 0.02 °2θ and 0.2 s per step. The measurement was performed in the range of 10° to 60° in the 2θ range.

As has been previously reported,[4,5] the decrease of interplanar distances follows the substitution of the $NH_2CH{=}NH_2^+$ (FA) cation for the smaller $CH_3NH_3^+$ (MA) cation in the perovskite unit cell. The reduction of interplanar distances shifts the position of the peaks corresponding to the cubic face toward higher 2θ angles in the XRD diffraction patterns of the characterized samples with variable proportions of FA and MA (Figure S2).

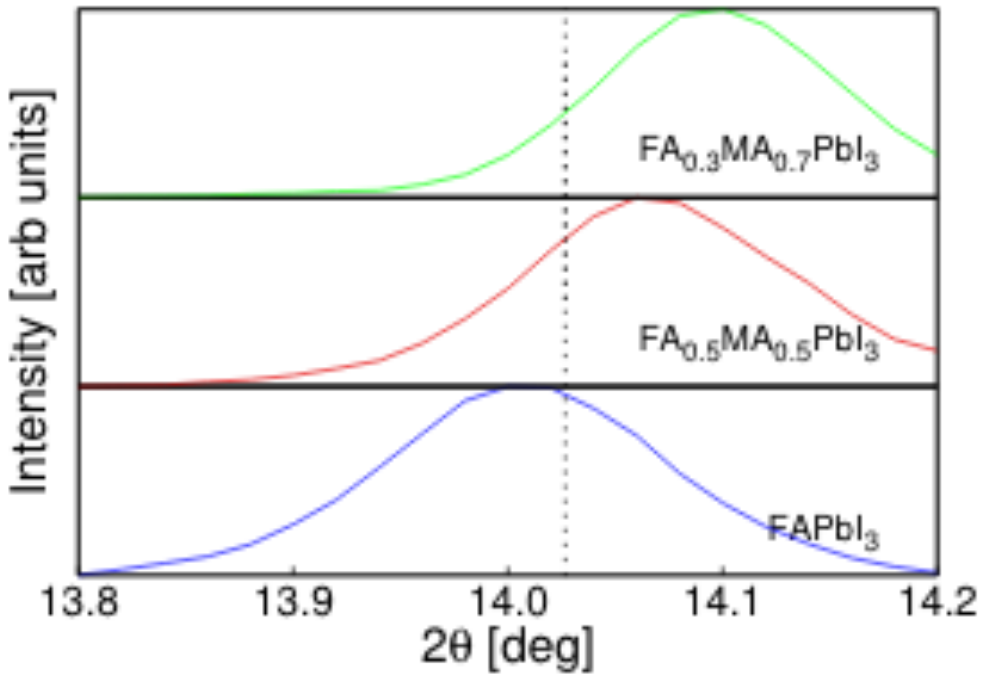

Figure S3. Simulated (vertical dotted line) and experimental results of the position of the peak that represents the α-black phase of FA perovskite when increasing the MA/FA ratio.

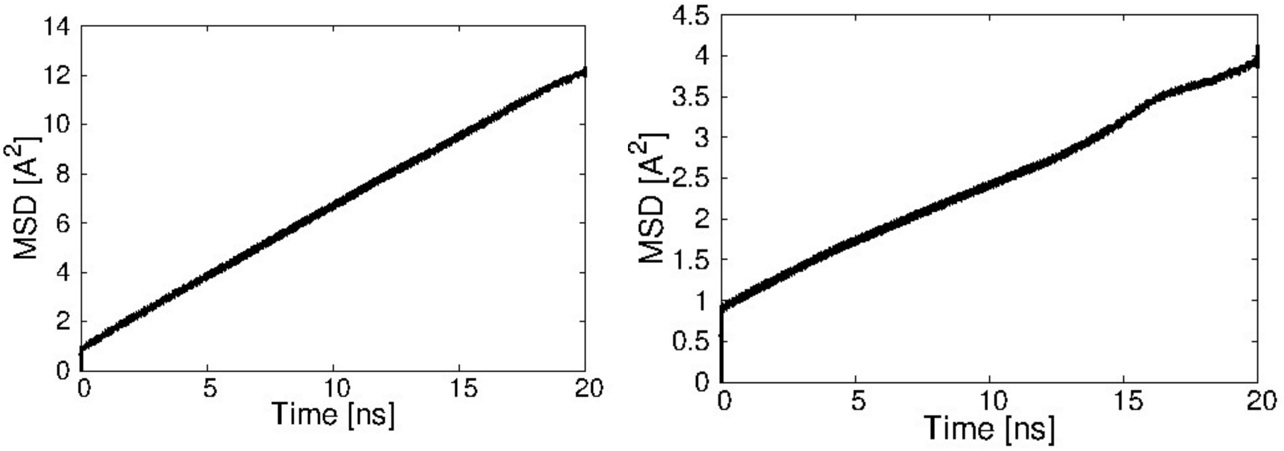

Figure S4. MSDs for Br diffusion in $MAPbBr_3$ and $FAPbBr_3$

References

(1) Hata, T.; Giorgi, G.; Yamashita, K.; Caddeo, C.; Mattoni, A. Development of a Classical Interatomic Potential for MAPbBr3. *J. Phys. Chem. C* 2017, *121* (7), 3724–3733. https://doi.org/10.1021/acs.jpcc.6b11298.

(2) Mattoni, A.; Filippetti, A.; Caddeo, C. Modeling Hybrid Perovskites by Molecular Dynamics. *J. Phys. Condens. Matter Inst. Phys. J.* 2017, *29* (4), 043001. https://doi.org/10.1088/1361-648X/29/4/043001.

(3) Eames, C.; Frost, J. M.; Barnes, P. R. F.; O'Regan, B. C.; Walsh, A.; Islam, M. S. Ionic Transport in Hybrid Lead Iodide Perovskite Solar Cells. *Nat. Commun.* 2015, *6*, 7497. https://doi.org/10.1038/ncomms8497.

(4) Yuan, D.-X.; Gorka, A.; Xu, M.-F.; Wang, Z.-K.; Liao, L.-S. Inverted Planar NH2CHNH2PbI3 Perovskite Solar Cells with 13.56% Efficiency via Low Temperature Processing. *Phys. Chem. Chem. Phys.* 2015, *17* (30), 19745–19750. https://doi.org/10.1039/C5CP02705E.

(5) Jacobsson, T. J.; Correa-Baena, J.-P.; Pazoki, M.; Saliba, M.; Schenk, K.; Grätzel, M.; Hagfeldt, A. Exploration of the Compositional Space for Mixed Lead Halogen Perovskites for High Efficiency Solar Cells. *Energy Environ. Sci.* 2016, *9* (5), 1706–1724. https://doi.org/10.1039/C6EE00030D.
